# Microbial enhancement of plant nutrient acquisition

**DOI:** 10.1007/s44154-021-00027-w

**Published:** 2022-01-10

**Authors:** Sunil K. Singh, Xiaoxuan Wu, Chuyang Shao, Huiming Zhang

**Affiliations:** 1 Shanghai Center for Plant Stress Biology, Center for Excellence in Molecular Plant Sciences, Chinese Academy of Sciences, Shanghai, 201602 China; 2 University of Chinese Academy of Sciences, Beijing, 100049 China

**Keywords:** Beneficial microbes, Plant, Macronutrient, Micronutrient, Plant-microbe interactions, Volatile organic compounds, Microbiome

## Abstract

Nutrient availability is a determining factor for crop yield and quality. While fertilization is a major approach for improving plant nutrition, its efficacy can be limited and the production and application of fertilizers frequently bring problems to the environment. A large number of soil microbes are capable of enhancing plant nutrient acquisition and thereby offer environmentally benign solutions to meet the requirements of plant nutrition. Herein we provide summations of how beneficial microbes enhance plant acquisition of macronutrients and micronutrients. We also review recent studies on nutrition-dependent plant-microbe interactions, which highlight the plant’s initiative in establishing or deterring the plant-microbe association. By dissecting complex signaling interactions between microbes within the root microbiome, a greater understanding of microbe-enhanced plant nutrition under specific biotic and abiotic stresses will be possible.

## Introduction

In addition to the elements carbon (C), hydrogen (H), and oxygen (O) that can be provide from CO_2_ and water, terrestrial plants commonly require a list of elemental nutrients from the soil to support healthy growth and development. These essential nutrients include nitrogen (N), phosphorus (P), potassium (K), sulfur (S), magnesium (Mg), and calcium (Ca), which are collectively termed macronutrients due to the relatively large quantities required for plants, as well as iron (Fe), manganese (Mn), copper (Cu), zinc (Zn), boron (B), molybdenum (Mo), chlorine (Cl), and nickel (Ni), which are collectively termed micronutrients due to the relatively small plant requirement. Plant deficiency in each of the macronutrients and micronutrients leads to varying consequences at molecular and phenotypic levels (Andresen et al. 2018, Gong et al. 2020).

Deficiency of one or more nutrients available for plant utilization is common in natural soils. For example, only a very small fraction of P in soils and bedrocks is potentially available to plants (Alewell et al. 2020), meanwhile nearly 50% of the rice-growing soils are Zn-deficient (Krithika and Balachandar 2016). Fertilization is a major approach for improving plant nutrition. However, the efficacy of fertilization in agriculture can be limited by environmental interferences, as exemplified by Zn fertilization with the water soluble zinc sulfate, which is often precipitated as hydroxides, carbonates, phosphates and sulfides in the soil and consequently shows only 1–5% fertilizer use efficiency (Krithika and Balachandar 2016). More importantly, the production and application of fertilizers in global agriculture have posed serious problems to the environment. For instance, industrial production of N fertilizers consumes 1–2% of the world’s fossil fuel energy output (Chen et al. 2018), meanwhile plants take up only 30–50% of N available in the soil and the remaining N fertilizers are lost through nitrification or denitrification processes, which cause eutrophication and other hazardous impacts to soil, water, and the atmosphere (Lehnert et al. 2018). Similarly, the agricultural usage of industrial P fertilizer has been not only increasingly consuming non-renewable geological P deposits, but also making major contributions to the eutrophication of fresh and costal water bodies around the world (Smil 2000, Alewell et al. 2020).

**Fig. 1:**
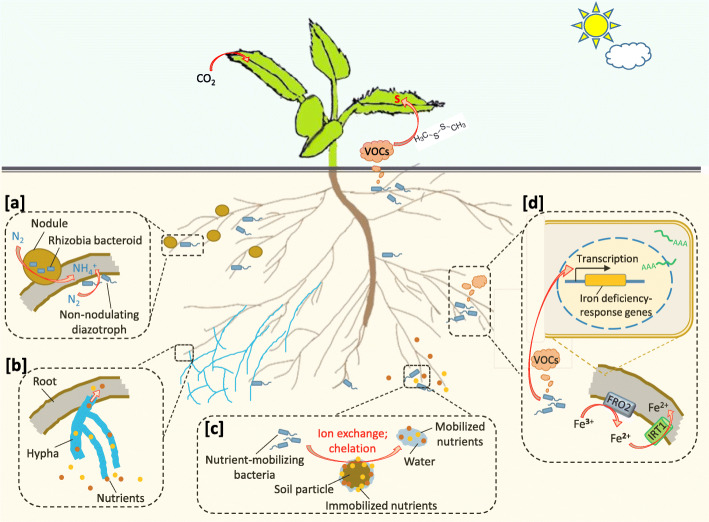
Microbe-enhanced plant acquisition of macronutrients and micronutrients. Beneficial microbes enhance plant nutrient acquisition via multiple mechanisms, including but not limited to [**a**] N_2_ fixation by rhizobia in nodules or by non-nodulating diazotrophs; [**b**] Nutrient uptake and delivery through mycorrhizal mycelia that reaches additional soil beyond the root; [**c**] Mobilization of soil-fixed nutrients through ion exchange or chelation by bacterial or fungal secretions, such as organic acids and siderophores; [**d**] Microbe-induced transcriptional regulation of plant genes involved in nutrient uptake, such as Arabidopsis Fe deficiency responses that are induced by bacteria volatile organic compounds (VOCs). Microbes may also enhance plant S nutrition via certain S-containing VOCs, such as dimethyl disulfite, which can be assimilated by the aerial portion of plants

Plants naturally live with a plethora of soil microbes, some of which are beneficial in that they can promote plant growth and/or increase plant resistance to one or multiple stress conditions, including nutrient deficiency (Kloepper et al. 1980, Lugtenberg and Kamilova 2009; Morcillo and Manzanera, 2021). Within the rhizosphere where root exudates provide an environment enriched with organic nutrients, beneficial microbes can be free-living or directly associated with plants as epiphytes or endophytes; particularly, plant-associated fungi extend mycelia to additional soil beyond the rhizosphere and can therefore reach for more nutrients to support the association (Fig. 1). These beneficial microbes provide an environment-friendly approach for improving plant nutrition, either as enhancers or alternatives to chemical fertilizers. Some of the beneficial microbes, such as rhizobia that fix atmospheric nitrogen, are well-known and have been applied in agriculture, while many others remain to be characterized and exploited. Herein we review how beneficial microbes improve plant acquisition of the macronutrients N, P, K, Mg, and S as well as the micronutrients Fe, Mn, Cu, Zn, B, Mo, and Ni. We also review recent works that investigated nutrition-dependent plant-microbe interactions, which demonstrate the plant’s initiative in determining the plant-microbe association for optimized benefits including P nutrition.

## Microbial enhancement of plant macronutrient acquisition

### Nitrogen (N)

Microbes play significant roles in plant acquisition of ammonium (NH_4_^+^) and nitrate (NO_3_^−^). Diazotrophs are N_2_-fixing bacteria and archea with nitrogenase that catalyzes the reduction of atomospheric N_2_ to NH_3_, which is then ionized to NH_4_^+^ favoring low environmental pH. Ammonium is also generated by bacterial or fungal decomposition of organic debris and can be oxidized by nitrifying bacteria to nitric oxides and nitrates. Some N_2_-fixing bacteria are free-living in soil, while others are plant-associated or even require symbiosis with plants to fix N_2_ (Fig. 1a). Symbiotic N_2_ fixation occurs in root nodules, where the specialized environment ensures the bacterial fixation of N_2_ and the bacteroids receive plant-derived organic nutrients in exchange of NH_3_ (Mus et al. 2016, Roy et al. 2020). Plants capable of forming nodules mostly belong to the family of Leguminosae. These leguminous plants host species-specific symbiosis with rhizobia from the Rhizobiaceae family (Andrews and Andrews 2017). In addition to rhizobia-legume symbiosis, N_2_-fixing nodulation also occur in *Parasponia* species that host rhizobia and in actinorhizal plants that host the actinomycete *Frankia* (Svistoonoff et al. 2014, van Velzen et al. 2018).

Plant N acquisition can also be improved by non-nodulating diazotrophs. For instance, inoculation with the nitrogen-fixing cyanobacterium *Nostoc punctiforme* resulted in detectable nitrogenase activity in rice roots and promotion of plant growth under nitrogen deficiency (Álvarez et al. 2020). *Azospirillum brasilense* strain HM053, which fixes nitrogen and excretes NH_4_^+^ constitutively, was capable of colonizing the surface of wheat roots and substantially increasing the plant dry weight (Santos et al. 2017). In addition, horizontal transfer of the nitrogen fixation (*nif*) genes can enable the capacity of nitrogen fixation in non-diazotrophic microbes (Dixon and Postgate 1971, Jing et al. 2020). For instance, genetic engineering of the *nif* genes from a *Pseudomonas stutzeri* strain into a *Pseudomonas protegens* strain enables the latter the ability of nitrogen fixation and consequently causing plant growth-promotion under nitrogen deficiency (Jing et al. 2020).

Besides diazotrophs, mycorrhizal fungi are also well-known to improve plant N acquisition, because mycorrhizal mycelia reaches additional soil beyond the root for more nutrients and can transport the assimilated N to the roots in a mutualistic relation (Fig. 1b). By using stable isotope labelling of N, it has been shown that inorganic nitrogen taken up by arbuscular mycorrhizal (AM) fungus outside the roots is incorporated into amino acids, translocated from the extraradical to the intraradical mycelium as arginine and transferred to the plant without carbon (Govindarajulu et al. 2005). In addition, plant transporters for N acquisition can be up-regulated by fungal symbionts. For instance, colonization of the AM fungus *Rhizophagus irregularis* strongly induced gene expression of the putative nitrate transporter *OsNPF4.5* in rice roots and promoted plant growth with remarkable increases in N acquisition, whereas knockout of *OsNPF4.5* resulted in a 45% decrease in symbiotic N uptake and a significant reduction in arbuscule incidence when NO_3_^−^ was supplied as an N source (Wang et al. 2020). Given that most staple crops are non-legumes, these non-nodulating bacteria and fungi offer important tools for improving plant N nutrition in agriculture.

### Phosphorus (P)

The majority of natural P in soil are in insoluble form and thus unavailable to plants (Richardson and Simpson 2011). A wide variety of bacteria and fungi species are capable of solubilizing inorganic P and/or mineralizing organic P, thereby releasing bioavailable P that can be readily absorbed by plants (Fig. 1b, c). Strains from the bacteria genera *Pseudomonas*, *Bacillus* and *Rhizobium* are among the most powerful P-solubilizing microbes (Rodríguez and Fraga 1999). Immobilized inorganic P can be released by microbe-secreted organic acids, such as gluconic acid and citric acid. Anions from these organic acids chelate cations that otherwise precipitate P-containing anions (Whitelaw et al. 1999), meanwhile acidification of the soil environment releases P from precipitates through the formation of soluble hydrogen (HPO_4_^2**−**^) or dihydrogen (H_2_PO_4_^**−**^) phosphates (Sharma et al. 2013). Protons that causes acidification can also be secreted through H^+^-ATPase, through transporter-assisted cation exchange across the microbial membranes, or in the form of inorganic acids (Alori et al. 2017). Microbial mineralization of organic P is mainly catalyzed by non-specific acid phosphatases that dephosphorylate the phosphor-ester or phosphoanhydride bonds of organic molecules (Nannipieri et al. 2011), as well as by phytases that specifically hydrolyze phytate (*myo*-inositol hexakisphosphate) in releasing the orthophosphate anion (Mitchell et al. 1997, Lim et al. 2007). The hydrolysis of phytate by phytases produces *myo*-inositol (Mitchell et al. 1997), which is a common component of root exudates in many plant species (Vílchez et al. 2020). Phytate is the most abundant organic phosphorus compound in soil (Lim et al. 2007), while *myo*-inositol attracts *Bacillus megaterium* spp. that is well-known for strong P-solubilizing ability (Vílchez et al. 2020). Thus it is an interesting question whether the plant exudation of *myo*-inositol carries a function in attracting *B. megaterium* spp. for bacteria-assisted P acquisition from the hydrolysis of phytate.

In addition to providing more bioavailable P to plants, beneficial microbes can also stimulate plant P uptake through regulation of plant P transporters. For instance, colonization of AM fungi significantly increased the transcript level of LePT4, a phosphate transporter in tomato plants, with a concomitant 2-fold increase in phosphate uptake; meanwhile a loss-of-function mutation in *LePT4* significantly impaired the AM fungi-enhanced phosphate uptake (Xu et al. 2007). LePT4 belongs to the Pht1 family of plant phosphate transporters, which are expressed at both the root–soil interface and the symbiotic fungus–plant interface around arbuscules or hyphal coil (Nagy et al. 2005). Phosphate transporter genes specifically activated in AM fungi symbiosis have been identified in many agricultural plant species, such as potato (*Solanum tuberosum*), rice (*Oryza sativa*), and *Medicago truncatula* (Rausch et al. 2001, Harrison et al. 2002, Paszkowski et al. 2002).

Fungi may be considered as more effective tools than bacteria in promoting plant P acquisition, because the former release more organic acids, reach more resources, and are capable of transmitting phosphate to the symbiotic plant cells (Sharma et al. 2013). However, a combination of P-solubilizing bacteria and fungi can result in better effects than bacteria or fungi alone. For instance, a combinational inoculation with AM fungi and beneficial rhizobacteria to durum wheat increased transcript levels of the P transporters Pht1 and PT2–1 as well as the aboveground P contents; in contrast, AM fungi alone induced Pht2 gene expression and required additional supply of organic N to similarly increase plant P contents (Saia et al. 2015). Recently it was shown that the extraradical hyphae of AM fungi can transport P-solubilizing bacteria to organic P patches and enhance organic P mineralization both under in vitro culture and soil conditions, in a way that hyphal exudates are required as the energy source for transferring the bacteria (Jiang et al. 2021), thus supporting the advantage of using fungi and bacteria in combinations for enhancing P acquisition in plants.

### Potassium (K)

In most soils, the solution and exchangeable K constitute only a few percent of the total soil K (Brady et al. 2008). Many bacterial and fungal strains have been documented as capable of releasing K from silicate minerals such as mica, biotite, and orthoclase (Meena et al. 2014, Masood and Bano 2016). These microbes enhance mineral weathering under in vitro conditions and increase K solubilization in soil, mainly through acidolysis, chelation and exchange reactions, in which microbe-secreted organic acids play an important role (Masood and Bano 2016, Meena et al. 2014). K enrichment resulted from AM fungi colonization has been demonstrated in different plant organs such as *Zea mays* root steles, *Pelargonium peltatum* shoots, and *Lactuca sativa* leaves (Perner et al. 2007, Baslam et al. 2013).

Many of the K-solubilizing microbes are saprophytic strains (Meena et al. 2014). Colonization by *Clitopilus hobsonii*, a common soil saprophytic fungus, increased plant growth and facilitated K uptake in the American sweetgum (*Liquidambar styraciflua*), particularly under K limitation conditions (Peng et al. 2021). Gene expression of ChACU, a putative K^+^ transporter in *C. hobsonii*, were detected in the colonized roots together with two other similar genes and were up-regulated by low K^+^ conditions; however, the expression of plant genes related to K^+^ uptake was not altered by the symbiosis with *C. hobsonii* (Peng et al. 2021). Thus the *C. hobsonii* K^+^ transporters appear to play a more active role than the sweetgum K^+^ transporters in the symbiotic supply of K. In contrast, fungal induction of plant putative K^+^ transporter genes was observed in perennial ryegrass (*Lolium perenne*) colonized by *Aspergillus aculeatus*, concomitant with higher levels of soluble K contents (Li et al. 2021). In another symbiosis example, the ectomycorrhizal fungus *Hebeloma cylindrosporum* significantly improved the K nutrition of pine (*Pinus pinaster*) plants under potassium-limiting conditions, and the fungal tandem-pore outward-rectifying K^+^ (TOK) channels are likely responsible for releasing K^+^ from the fungal Hartig net to the plant (Guerrero-Galán et al. 2018). The efficacy of microbial enhancement of plant K nutrition can be improved by co-application with K-containing minerals, as shown in perennial ryegrass treated with *A. aculeatus* plus K-containing feldspar compared with plants treated with the fungus alone (Li et al. 2021).

### Sulfur (S)

Plant sulfur nutrition depends primarily on the uptake of inorganic sulfate (SO_4_^2−^), whereas more than 95% of the soil sulfur in agricultural soils is present as sulfate esters or as carbon-bonded sulfur in the forms of sulfonates and amino acid sulfur (Kertesz and Mirleau 2004, Zhao et al. 2006). Many bacterial and fungal species in the rhizosphere are capable of releasing S from sulfate esters, as a result of the mineralization catalyzed by sulfatases, which include arylsulfatase that cleaves the O-S bond in aromatic sulfate-esters and alkylsulfatase that cleaves the C-O bond in aliphatic sulfate-esters (Kertesz 2000). In contrast to the mineralization of sulfate esters, releasing S from sulfonates is catalyzed by a multicomponent mono-oxygenase enzyme complex encoded in the *ssu* gene cluster (Eichhorn et al. 1999), which is found in bacteria but not fungi (Gahan and Schmalenberger 2015). The ability of releasing S from aliphatic sulfonates is widespread in soil bacteria (King and Quinn 1997), whereas releasing S from aromatic sulfonates requires the *asfRABC* gene cluster in addition to the *ssu* gene cluster (Vermeij et al. 1999). Aliphatic and aromatic sulfonates together account for 30–70% soil S (Zhao et al. 2006). It was suggested that aromatic sulfonates are more important than aliphatic sulfonates in microbe-assisted plant S nutrition, because bacterial ability in utilizing the former but not the latter has been associated with plant growth-promotion (Kertesz and Mirleau 2004, Gahan and Schmalenberger 2015). Although fungi are unable to mineralize sulfonates, some saprotrophic fungi may facilitate bacterial mineralization of sulfonates through depolymerizing polymeric sulfonates into monomeric or oligomeric sulfonates (Gahan and Schmalenberger 2015, Kertesz et al. 2007).

In addition to increasing the SO_4_^2−^ supply, soil microbes can also enhance plant S nutrition through transcriptional regulation of plant sulfate assimilation pathway. In *Arabidopsis thaliana* exposed to the volatiles organic compounds (VOCs) released from *Bacillus amyloliquefaciens* GB03, transcript levels of several genes, such as ATP sulfurlyase (ATPS) and adenosine 5′ –phosphosulfate reductase (APR), in the sulfate assimilation pathway were up-regulated; concomitantly, VOC-enhanced plant sulfate uptake was observed with radio-labeled sulfate within 30 min (Aziz et al. 2016). In *Lotus japonicas* colonized by the AM fungus *R. irregularis*, gene expression of the sulfate transporter LjSultr1;2 was localized in arbuscule-containing cells and up-regulated by either the fungal colonization or S starvation (Giovannetti et al. 2014). Dysfunction of LjSultr1;2 decreased plant constitutive S uptake, while the fungal colonization increased plant sulfate contents under S starvation (Giovannetti et al. 2014), thus supporting the importance of transcriptional regulation in the microbe-assisted plant S nutrition.

Unlike other microbes that enhance plant S uptake in roots, *Bacillus* sp. B55 is capable of increasing plant sulfur acquisition in the air (Meldau et al. 2013). With radio-labeled sulfur ^35^S supplemented into the bacteria growth medium, B55 VOCs was shown to transmit sulfur to *Nicotiana attenuata* plants, which were grown in medium physically separated from the bacteria growth medium, and rescued plant growth retardation caused by sulfur starvation. Dimethyl disulfide (DMDS) was identified as the major S-containing component in B55 VOCs and responsible for the VOC-enhanced plant S nutrition (Meldau et al. 2013). Sulfur in SO_4_^2−^ is in an oxidative state that needs an energy-consuming reduction process for biological assimilation (Takahashi et al. 2011), whereas sulfur in DMDS is in a chemically reduced state, which may credit DMDS as an energy-saving form of sulfur nutrition to plants. Consistent with this hypothesis, sulfur assimilation as well as methionine biosynthesis and recycling in plants were transcriptionally repressed by DMDS, indicating a decreased demand for SO_4_^2−^ reduction (Meldau et al. 2013). DMDS is a common component in microbial VOCs; meanwhile other microbes can produce VOCs with high levels of other sulfur-containing compounds such as dimethyl sulfide and dimethyl trisulfide (Kanchiswamy et al. 2015). Therefore, VOC-mediated sulfur assimilation in the air might be a common mechanism for microbe-enhanced plant S nutrition.

### Magnesium (Mg)

Plants grown in acidic and sandy soils frequently suffer from Mg deficiency due to leaching and antagonism by other cations (Hermans et al. 2010). A few examples have been reported for microbe-mediated alleviation of plant Mg deficiency. In *A. thaliana* particularly under Mg deficiency condition, the plant Mg nutrition was improved by the colonization of *Piriformospora indica*, a root endophytic fungus that possesses the Mg transporter PiMgT1 (Prasad et al. 2019). Inoculation of AM fungus *Funneliformis mosseae* to *Poncirus trifoliata* seedlings resulted in enhanced growth promotion and suppression of Mg deficiency symptoms; the reduction in Mg deficiency symptoms may be attributed to the fungus-induced elevations in whole-plant soluble protein levels and in the activity of root catalase and superoxide dismutase (Zhang et al. 2015). Interesting, the colonization of *F. mosseae* to *P. trifoliata* roots was significantly higher under Mg-deficiency than under Mg-sufficiency (Zhang et al. 2015), whereas sweet potato and onion leaves sprayed with high levels of MgSO_4_ displayed strongly inhibited root AM fungus colonization and sporulation in aeroponic and sand culture (Jarstfer et al. 1998), indicating a negative correlation between plant Mg availability and AM fungi colonization to roots.

## Microbial enhancement of plant micronutrient acquisition

### Iron (Fe)

Plants use different strategies to increase iron mobility in the rhizosphere. Graminaceous monocots secret siderophores to chelate ferric iron (Fe^3+^) before its root uptake, while non-graminaceous monocots and dicots deploy a combined strategy including rhizosphere acidification, reduction of Fe^3+^ to ferrous iron (Fe^2+^) by plasma membrane ferric reductase, and transporter-mediated Fe^2+^ import into roots (Curie and Briat 2003). These strategies are involved in microbial enhancement of plant iron acquisition (Fig. 1d).

Microbial siderophores in their Fe-bound forms can be directly absorbed by plants. Hydroxymates and catechols are the major functional groups in the iron-siderophore binding (Rajkumar et al. 2010). Siderophores produced from different microbes can be characterized by their structural differences, for instance, siderophores in the form of rhizobactin (catechol-containing) and citrate (neither a catechol nor a hydroxamate) are released from *Rhizobium meliloti* and *Bradyrhizobium japonicum*, respectively (Smith et al. 1985, Guerinot et al. 1990). In addition to providing microbial siderophores, certain microbes also stimulate plant production of phytosiderophores. In sorghum (*Sorghum bicolor*) under Fe deficiency, gene expression of SbDMAS2 (deoxymugineic acid synthase 2), SbNAS2 (nicotianamine synthase 2), and SbYS1 (Fe-phytosiderophore transporter yellow stripe) was induced by AM fungus, concomitant with significant increases in phytosiderophore release and Fe uptake in the plant (Prity et al. 2020).

Acidification improves Fe^3+^ mobility, thus, rhizosphere acidification stimulated or directly contributed by microbes facilitates plant iron acquisition. Organic acids commonly exist in microbial extracellular metabolites. For instance, the VOCs from *B. amyloliquefaciens* GB03 contain glyoxylic acid, 3-methyl-butanoic acid and diethyl acetic acid (Farag et al. 2006), which directly causes rhizosphere acidification (Zhang et al. 2009). GB03 VOCs also stimulate plant acidification of the rhizosphere, as shown by VOC-exposed Arabidopsis that were transferred to new growth medium with no VOCs anymore (Zhang et al. 2009). Importantly, Arabidopsis under Fe-sufficient conditions responded to GB03 VOCs with systemic Fe-deficiency responses, including transcriptional induction of the Fe^3+^ reductase FRO2 and the Fe^2+^ transporter IRT1, as well as elevated enzyme activity of FRO2, in addition to the stimulation of rhizosphere acidification by the plant (Zhang et al. 2009). As a result, Fe levels were elevated in the VOC-exposed plants (Zhang et al. 2009), in supporting plant production of greater amounts of Fe-containing photosynthetic apparatus (Zhang et al. 2009, Zhang et al. 2008). The phenotype and/or mechanism of GB03 VOC-enhanced plant Fe acquisition were similarly observed in many later studies, including investigations on other plants species, VOCs from other microbes, and soil inoculation with GB03 or other beneficial microbes (del Carmen Orozco-Mosqueda et al. 2013a, del Carmen Orozco-Mosqueda et al. 2013b, Castulo-Rubio et al. 2015, Freitas et al. 2015, Zamioudis et al. 2015, Zhou et al. 2016, Martínez-Medina et al. 2017, Hernández-Calderón et al. 2018, Montejano-Ramírez et al. 2020, Kong et al. 2021). Therefore, microbial regulation of plant Fe-deficiency responses plays a prominent role in microbe-enhanced plant Fe acquisition (Fig. 1d).

The volatile compound *N*,*N*-dimethylhexadecylamine (DMHDA), which is commonly produced by several bacteria strains belonging to *Arthrobacter agilis*, *Sinorhizobium meliloti*, or *Pseudomonas fluorescens*, was shown to induce plant Fe-deficiency responses (del Carmen Orozco-Mosqueda et al. 2013a, Castulo-Rubio et al. 2015, Montejano-Ramírez et al. 2020). GB03 VOCs’ effects on plant Fe-acquisition requires transcriptional induction of FIT1 (Zhang et al. 2009), a master regulator of Fe-deficiency responses conserved in plants (Riaz and Guerinot 2021). The bioactive VOC component(s) responsible for this key regulatory step along with its mechanism have been elusive. Although identifying the bioactive component or combination from natural microbial VOC blends has been challenging, the beneficial effects of microbial VOCs on plant resistance to biotic and abiotic stress have been accumulating (Farag et al. 2013, Liu and Zhang 2015, Weisskopf et al. 2021), making these microbial metabolites important resources for developing new tools for agricultural applications.

### Copper (Cu)

AM fungi are capable of increasing plant fitness under Cu deficient and toxic conditions (Ferrol et al. 2016). In white clover (*Trifolium repens* L.) plants colonized by *Glomus mosseae*, the improvement in plant Cu nutrition was attributed to extraradical mycelia that extend out of the root into the surrounding environment for more nutrients (Li et al. 1991). Similarly, *G. mosseae* colonization increased Cu contents in the shoots of cucumber (*Cucumis sativus*), owing to extraradical mycelia-dependent nutrient uptake (Lee and George 2005). In the mycorrhizalwhite clover, the fungal contribution to plant Cu uptake was independent of P availability; however, increases in exogenous P supplies from soil changed the distribution patterns of the Cu contents contributed by *G. mosseae*, with a decrease and an increase in the roots and the shoots, respectively (Li et al. 1991). Similarly, in the mycorrhizal cucumber plants, high levels of P supply to hyphae resulted in decreased root Cu concentrations (Lee and George 2005), thus drawing attentions to the crosstalk among different nutrient improvements in mycorrhizal plants.

Bacterial improvements of plant Cu nutrition have been correlated with microbial siderophores or modulation of certain plant genes. In alfalfa (*Medicago sativa*) seedlings, the Cu and Fe uptake was enhanced by two siderophores producing bacteria strains of *P. fluorescens* and *Rhizobium leguminosarum* bv *phaseoli* (Carrillo-Castañeda et al. 2002). In cucumber plants under Cu deficiency or dual deficiency of Cu and Fe, plant stress symptoms were alleviated by the plant-beneficial bacteria *A. brasilense*, accompanied by better root development and nutrient uptake (Marastoni et al. 2019). In consistence with the bacteria-enhanced plant nutrient uptake, *A. brasilense*-induced *CsFRO* gene expression was observed in the cucumber plants, which take up Cu after FRO-mediated reduction (Marastoni et al. 2019).

### Manganese (Mn)

Plants acquire Mn only in its divalent form (Mn^2+^). Mn is poorly available for plant uptake in well-aerated calcareous soils, because alkaline and oxidative conditions favor the formation of water-insoluble Mn oxides; meanwhile Mn^2+^ soil dressing is rather ineffective to alleviate deficiency due to rapid oxidation of Mn^2+^ (Andresen et al. 2018). Since Mn availability in soils is determined by pH, microbe-mediated rhizosphere acidification would improve plant Mn acquisition. Examples of bacterial mobilization of Mn can be seen in some acidophilic bacteria strains, such as *Acinetobacter* sp. and *Lysinibacillus* sp., which were used for Mn bioleaching from ores or mining waste water (Sanket et al. 2017, Ghosh et al. 2018). Similarly, Mn solubilizing fungal strains, such as *Aspergillus terreus* and *Penicillium daleae*, have been isolated from low-grade Mn mine tailings, and their Mn solubilizing ability was attributed to the mycelia production of organic acids such as oxalic acid, citric acid, maleic acid and gluconic acid (Mohanty et al. 2017).

Due to its low selectivity, the Fe^2+^ importer IRT1 is also one of the Mn transporter in plants (Barberon et al. 2014). In addition, the availability of both Fe and Mn in soil are determined by pH and redox potential. Thus, plant Mn uptake may be enhanced by microbes that are capable of triggering plant iron deficiency responses. In a calcareous soil, the rhizosphere microbiome of maize (*Z. mays*) harbored populations of Fe^3+^-reducing microbes and Mn^4+^-reducing microbes, with the latter being one magnitude greater than the former (Kothari et al. 1991). Consistent with this Mn^4+^-reducing potential, plants grown in natural soil accumulated higher levels of Mn than plants grown in sterilized soil. Interestingly, adding the AM fungus *G. mosseae* to the non-sterilized soil almost depleted the populations of Mn^4+^-reducing microbes and Fe^3+^-reducing microbes, while the rest of the total microbial population remained basically unchanged in terms of quantity; concomitantly, the native microbiome-dependent enhancement of plant Mn accumulation was substantially decreased by the fungus, although the mycorrhizal plants still showed mild increases in plant Mn levels, which likely resulted from the fungus-assisted uptake (Kothari et al. 1991). These observations demonstrated the ability of rhizosphere microbiome in enhancing plant Mn acquisition. In addition, the impacts of *G. mosseae* on the Mn^4+^-reducing and Fe^3+^-reducing microbes highlight the importance of considerations on native microbiome when applying microbes for plant benefits.

When applying microbes capable of enhancing Mn availability to plants, cautions are required since Mn in excess imposes ionic stress to plant cells. For instance, in soybean (*Glycine max* L.) with abundant soil Mn availability, inoculation with the AM fungus *Glomus macrocarpum* resulted in strong Mn accumulation, Mn toxicity symptoms and reduced biomass in comparison to control plants (Nogueira and Cardoso 2003). Although *G. macrocarpum* enhanced plant growth under low Mn availability conditions, the mycorrhizal plants showed lower Mn contents in both shoots and roots compared to the control plants, meanwhile the P contents in the mycorrhizal plants were increased (Nogueira and Cardoso 2003). In contrast to *G. macrocarpum*, two *G. etunicatum* and *G. intraradices* strains promoted plant growth with elevated plant P contents under both low and high soil Mn availability, even though mild increases in Mn contents occurred in the mycorrhizal plants with high Mn availability (Nogueira and Cardoso 2003). Therefore, evaluations on microbial efficacy in increasing plant Mn acquisition would help choose an optimized strain for broad-ranging usages.

### Zinc (Zn)

Zn deficiency frequently impairs the yield and quality of staple food crops. It was estimated that about 50% of the rice-growing soils are under Zn-deficient, and that Zn fertilization by using the soluble ZnSO_4_ typically result with very low fertilizer use efficiency (1–5%) due to Zn immobilization in the soil (Krithika and Balachandar 2016). Zn-mobilizing microbes offer alternative tools for enhancing plant Zn acquisition. In rice (*O. sativa*) seedlings, comparable effects on plant Zn accumulation were observed between the ZnSO_4_ fertilization and the application of insoluble Zn oxide bound with Zn-mobilizing bacteria (Krithika and Balachandar 2016). In another experiment with natural soil deficient in Zn, application of Zn-mobilizing bacteria resulted in better effects in increasing plant Zn contents than Zn fertilization at the rate of 2.5 mg Zn as ZnSO_4_ .7H_2_O per kg soil (Vaid et al. 2014). A variety of bacteria such as *Pseudomonas* and *Bacillus* strains are known to enhance plant growth with higher Zn contents, and the microbial mobilization of Zn were attributed to different mechanisms mediated through rhizosphere acidification, sequestration by siderophores or anions from organic acids, and oxido-reductive systems on cell membranes (Kamran et al. 2017). Fungi-assisted plant Zn acquisition is also evident. In mycorrhizal bread wheat (*Triticum aestivum*) and barley (*Hordeum vulgare*), the fungal contribution of plant Zn uptake was measured as up to 24.3% and 12.7% of total above-ground Zn in wheat and barley, respectively (Coccina et al. 2019). In mycorrhizal maize, the plant physiological function such as photosynthesis in Zn deficient soils was improved with better nutrient uptake (Saboor et al. 2021).

In addition to providing plants with mycorrhizal Zn supply, fungi and bacteria may also regulated plant Zn transporters. Inoculation of barley with the AM fungus *R. irregularis* resulted in significant gene up-regulation of HvZIP13, a Zn transporter gene that is induced by low Zn availability, concomitant with improved grain and straw Zn concentrations (Watts-Williams and Cavagnaro 2018). Similarly, *R. irregularis* inoculation increased plant tolerance to soil Zn deficiency in *M. truncatula*; concomitantly, the fungus induced gene expression of MtZIP5 that is inducible by Zn deficiency (Nguyen et al. 2019). In rice plants inoculated with the Zn-mobilizing bacteria *Enterobacter cloacae*, gene expression of OsZIP1 and OsZIP5 was induced in the absence of Zn oxide as the insoluble Zn source in the growth medium, whereas expression of these two genes as well as OsZIP4 was repressed by the bacteria in the presence of Zn oxide (Krithika and Balachandar 2016). The microbial repression of these ZIP genes may reflect a feedback regulation by the microbe-dependent Zn sufficiency, which is similarly observed in the *R. irregularis-*inoculated barley and Medicago (Watts-Williams and Cavagnaro 2018, Nguyen et al. 2019).

Importantly, while AM fungi are capable of improving plant Zn nutrition under low soil Zn conditions, fungal protection of plants from excessive Zn accumulation at high Zn availability have also been documented in different plant species such as red clover (*Trifolium pretense*), Medicago, and tomato (Li and Christie 2001, Watts-Williams et al. 2013, Nguyen et al. 2019). These dual plant-beneficial effects make AM fungi great choices as microbial tools for optimizing crop Zn acquisition.

### Molybdenum (Mo)

Plants take in Mo in the form of molybdate (MoO_4_^2−^). Apart from Cu, Mo is the least abundant essential micronutrient found in most plant tissues (Kaiser et al. 2005). Mo deficiencies are considered as rare in most agricultural cropping areas, meanwhile its fertilization through foliar sprays can effectively supplement internal Mo and rescue the activity of Mo-dependent enzymes (Kaiser et al. 2005). Nonetheless, plant Mo acquisition can be improved by certain microbes. In sweet sorghum grown in an agricultural soil spiked with different concentrations of MoS_2_, AM fungal colonization by a *Claroideoglomus etunicatum* strain significantly enhanced plant Mo concentrations in both shoots and roots (Shi et al. 2020). In maize plants growing in soil supplemented with different levels of (NH_4_)_2_MoO_4_, the same *C. etunicatum* strain also enhanced plant Mo concentrations in shoots and roots, with reductions in the shoot-to-root Mo ratio when Mo was supplemented at the levels considered as moderate and severe pollution (Shi et al. 2018).

### Nickel (Ni)

Ni is required in higher plants only by the enzyme urease, while the mechanism of its assimilation by plants is largely elusive (Andresen et al. 2018). This micronutrient is generally available in fertilized soil in form of additives to fertilizers. For example, rock phosphate, which is a raw material for phosphatic fertilizers, is known to contain Ni ranging between 16.8 to 50.4 mg kg^− 1^ (Chauhan et al. 2008). In fact, Ni has drawn much attention as a common heavy metal pollutant in soil, air, and water. Consequently, the research of microbes for improving plant Ni homeostasis has been focused on microbe-induced plant stress-alleviation, which is commonly mediated through reducing heavy metal uptake and/or increasing plant tolerance to the metal ion toxicity.

### Boron (B)

Soil B exists in the form of the uncharged boric acid that can be readily taken up by plants (Miwa et al. 2009). Although B deficiency in many crops reportedly occur globally, it can be readily prevented and corrected by both soil and foliar applications (Shorrocks 1997). However, given that the range between B deficiency and toxicity to plants is very narrow (Matthes et al. 2020), beneficial microbes may be applied to improve B homeostasis in plants. For instance, soil supplementation of 25 μM and 100 μM B both increased plant photosynthesis efficiency under drought stress; however, the supplementation of 100 μM B, but not 25 μM B, caused a clear reduction in plant dry weight under well-watered condition (Quiroga et al. 2020). Inoculation with the AM fungus *R. irregularis* to these maize plants increased plant dry weight under both B-supplemented conditions compared to the non-mycorrhizal plants, irrespectively of the water availability. Although its mechanism remains unclear, this example demonstrates the ability of certain microbes in improving the efficacy of B supplementation for better plant growth.

## Nutrition-dependent plant-microbe association

Under nutrient deficient conditions, the changes in plant metabolism may lead to changes in the root exudates and consequently affect the community of root-associated microbes. A well-known example is N deficiency-induced production of flavonoids, which induce the species-specific rhizobial production of nodulation factors that in turn provoke deformation of the root hairs and nodule primordium formation (Relić et al. 1994, Coronado et al. 1995). Recent studies of different plant species also demonstrated root exudate-mediated re-assembly of rhizosphere microbiome in coping with plant nutrient deficiency. In maize under nitrogen deficient conditions, the plants not only up-regulated LRT1-mediated lateral root development, which presumably enhanced root interactions with the bacteria family *Oxalobacteraceae* that includes diazotrophic members, but also secreted flavones that increased the enrichment of *Oxalobacteraceae* in the rhizosphere, resulting in promoted plant N acquisition and growth (Yu et al. 2021). In Arabidopsis under Fe deficient conditions, the plants up-regulated gene expression of MYB72 that promotes root secretion of the antimicrobial coumarin scopoletin, leading to inhibition of the soil-borne fungal pathogens *Fusarium oxysporum* and *Verticillium dahlia* but not the beneficial rhizobacteria *Pseudomonas simiae* WCS417 and *Pseudomonas capeferrum* WCS358 (Stringlis et al. 2018), which are capable of enhancing plant iron acquisition (Zamioudis et al. 2015).

**Fig. 2:**
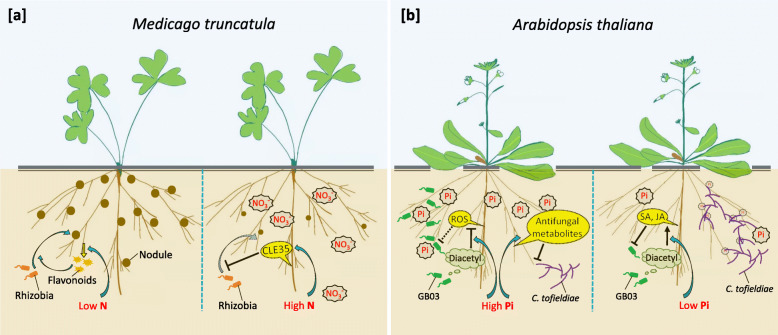
Nutrition-dependent plant-microbe association highlights plant’s initiative in determining the relationships. **a** Low N availability increases root production of flavonoids, which induce rhizobial production of nodulation factors that in turn provoke nodule formation for N_2_ fixation. By contrast, high N availability transcriptionally induces CLE35, which suppresses rhizobia infection and consequently nodulation (Moreau et al. 2021). **b** Under P sufficient conditions, Arabidopsis plants respond to the GB03-released volatile compound diacetyl with decreases in the microbe-induced ROS burst, thereby providing a permissive environment for the bacterial association; whereas under P deficient conditions, the plants respond to diacetyl with strong activation of salicylic acid (SA)- and jasmonic acid (JA)-mediated defense, thereby deterring the bacterial association (Morcillo et al. 2020). By contrast, Arabidopsis deploy a different strategy for determining the relation with *C. tofieldiae*, an endophytic fungus that can transfer phosphate to its host. The plants allow the symbiosis with *C. tofieldiae* only under P deficient conditions; whereas under P sufficient conditions, the plants deploy Trp-derived antifungal metabolites to deter the endophytic colonization of *C. tofieldiae* (Hiruma et al. 2016)

In contrast to the plant attraction of beneficial microbes for relieving nutrient deficiency, plant deterrence of the microbial association can occur under nutrient sufficient conditions (Fig. 2). This phenomenon can be seen in N fixation, which is inhibited by N fertilizers especially nitrate (Harper and Nicholas 1978). As shown in *M. truncatula*, this inhibition is mediated through MtCLE35, which is a CLAVATA3-like (CLE) signaling peptide that is transcriptionally up-regulated by local high N availability and that inhibits nodule formation (Moreau et al. 2021). Similarly, while mycorrhizal P uptake is a major benefit for plants colonized by AM fungi, sufficient P contents repress symbiotic gene expression and AM colonization in the roots of mycorrhizal petunia and tomato plants (Breuillin et al. 2010, Nagy et al. 2009). *A. thaliana* plants allow the symbiosis with *Colletotrichum tofieldiae*, an endophytic fungus that can transfer phosphate to its host, only under P deficient conditions; whereas under P sufficient conditions, the plants deploy Trp-derived antifungal metabolites to deter the endophytic colonization of *C. tofieldiae* (Hiruma et al. 2016). The P nutrition-dependent plant initiative was also observed in the association between Arabidopsis and the benefical rhizobacteria *B. amyloliquefaciens* GB03, albeit in an opposite manner compared to the association between Arabidopsis and *C. tofieldiae*. Under P sufficient conditions, the plants respond to the GB03-released volatile compound diacetyl with decreases in the microbe-induced ROS burst, thereby providing a permissive environment for the bacterial association; whereas under P deficient conditions, the plants respond to diacetyl with strong activation of salicylic acid (SA)- and jasmonic acid (JA)-mediated defense, thereby deterring the bacterial association (Morcillo et al. 2020). Thus Arabidopsis plants deploy different strategies in determining the association with fungi and bacteria. The different preferences on P availability probably reflect the different impacts by *C. tofieldiae* and GB03 on plant P acquisition, that is, the endophytic fungus can uptake and deliver P to the plant and is consequently welcomed by P-deficient plants, whereas the bacterium cannot deliver P to the plant and is consequently a competitor for P to P-deficient plants. Therefore, the nutrient-dependent alterations in plant-microbe interactions demonstrate the plant’s initiative in determining the plant-microbe association for optimized benefits (Fig. 2).

Activation of the SA- or JA-mediated defense augments plant P starvation responses (PSR) (Morcillo et al., 2020). This makes it risky for plants to simply deter the bacteria P competitors under P deficient conditions, if the plant cannot alleviate its P deficiency stress by taking up more P or by taking advantage of bacteria-released beneficial metabolites, which may be available in the rhizosphere without requiring bacteria colonization to the root. Indeed, when Arabidopsis plants were grown in P-depleted sterile medium with continuous exposure to diacetyl, the plants showed worsened PSR stress symptoms compared to their counterparts without exposure to diacetyl (Morcillo et al., 2020). Apparently, the binary interaction between the plant and the microbe is subject to the influence by many other biotic and abiotic factors. Plant interactions with individual microbes within the root microbiome are far more difficult to disentangle than in a binary system, because of the dynamic and interrelating correlations among the microbial community members and the plant. Consequently, nutrient-dependent changes in a particular microbiome member would likely reflect the outcome of a complex decision by the plant instead of a simple initiative based on the binary relation. For instance, soil-grown Arabidopsis with replete P supplementation showed increased abundance of *Olpidium brassicae*, a plant pathogen, in the root-associated fungal community (Fabiańska et al. 2019); meanwhile, medium-grown Arabidopsis treated with a bacterial synthetic community showed P deficiency-induced enrichment of *Burkholderia* spp., which exacerbated plant P starvation possibly through behaving as competitors to the plant for P nutrition (Finkel et al. 2019). It appears that these presumably unwelcomed microbes dominated in the relationships under the examined conditions; alternatively, the observations may indicate compromises for unidentified benefits in the plants.

## Conclusions and perspectives

A large number of microbes are capable of enhancing plant nutrition, mainly through increasing plant nutrient availability in soil and enhancing plant nutrient uptake (Fig. 1). Microbial regulation of plant genes for nutrient acquisition occurs, however, it remains largely unclear about what microbial factors induce the transcriptional regulation in the plant, and consequently about how the regulation is initiated at the molecular level. The beneficial microbes may also indirectly alleviate plant nutrient deficiency-stress, such as by increasing the activity of plant antioxidant enzymes to protect the plant from stress-induced ROS accumulation (Kabir et al. 2020). In addition, plant acquisition of some nutrients are inherently connected, indicating that beneficial microbes capable of enhancing plant acquisition of one nutrient may indirectly contribute to the acquisition of another nutrient. For instance, because Mg is important for rhizobial N2 fixation (Kiss et al. 2004), Mg supplementation to soybean plants improved the nodulation by *Bradyrhizobium* and the resultant N_2_ fixation under both N-deficient and N-sufficient conditions (Khaitov 2018). Thus, interesting questions exist such as whether legume plant N-acquisition can be enhanced by microbes that improve plant Mg acquisition, and whether legume plants would display a positive correlation between the activity of N_2_ fixation and the enrichment of Mg-mobilizing microbes in the root microbiome.

Beneficial microbes are valuable resources for developing environment-friendly tools to meet the nutrient requirements of crops with or without chemical fertilization. In using the beneficial microbes, some potential complications may be relatively easy to be envisioned, such as that a microbe beneficial to one plant species may be ineffective or even pathogenic to another, and that AM fungi capable of providing heavy metal micronutrients may either supply excessive ions or function as a protective barrier when the particular metal nutrient is replete in the environment. Meanwhile, our current understanding of the microbe-enhanced plant nutrient acquisition is largely simplified, because most studies investigated binary plant-microbe systems and were oftentimes under in vitro or artificial conditions, which are quite different from the complex and dynamic environmental conditions that plants would encounter in nature. Such a gap would be improved by research efforts in disentangling the crosstalk among various biotic and abiotic factors, as well as the microbe-microbe interactions within the root microbiome.

## Data Availability

Not applicable.

## Abbreviations

C: Carbon
H: hydrogen
O: oxygen
N: nitrogen
P: phosphorus
K: potassium
S: sulfur
Mg: magnesium
Ca: calcium
Fe: iron
Mn: manganese
Cu: copper
Zn: zinc
B: boron
Mo: molybdenum
Cl: chlorine
Ni: nickel
AM: arbuscular mycorrhizal
VOCs: volatile organic compounds
